# Autologous Non-cultured Basal Cell-Enriched Epidermal Cell Suspension Transplantation in Vitiligo: Indian Experience

**DOI:** 10.4103/0974-2077.79183

**Published:** 2011

**Authors:** Munish Paul

**Affiliations:** *Skin Laser Center, New Delhi, India*

**Keywords:** Vitiligo, cellular transplantation, autologous

## Abstract

**Introduction:**

Autologous non-cultured basal cell-enriched epidermal cell suspension transplantation is a simple yet effective cell-based therapy for vitiligo.

**Materials and Methods:**

This report is a retrospective analysis of 58 patients who were operated between December 2003 and August 2006 and were under follow-up for at least 2 years. Nine patients did not come for follow-up, and were excluded. At the time of transplantation all patients were having stable disease for at least 1 year. Epidermal cell suspension transplantation was done using Mulekar’s method. Repigmentation was assessed and classified into excellent (>90% repigmentation), good (70–89%), fair (30–69%) and poor (<30%).

**Results:**

Of the 49 patients who came for follow-up, 32 (65%) had excellent (>90%) repigmentation; 9 (18%) had good (70–89%); 4 (8%) had fair (30-69%) and 4 (8%) patients had poor (<30%) repigmentation. During the follow-up, eight patients(16%) showed relapse of the disease.

**Conclusion:**

Autologous noncultured basal cell-enriched epidermal cell suspension transplantation is an effective, simple and safe method.

## INTRODUCTION

Vitiligo is a common depigmentary disorder affecting about 2% of the world population regardless of race, ethnic background or gender. On the dark skin, these depigmented patches have a very striking and disfiguring effect causing many a times severe psychological problems including stress, low self-esteem, depression and suicidal tendencies.

For patients with stable disease, surgery is an option when medical therapies fail. In recent years, cellular transplantation such as the non-cultured melanocyte-keratinocyte suspension has gained popularity because of minimal technical complexity, superior aesthetic results and requirement of only a small donor area. We hereby report our experience with this technique.

## MATERIALS AND METHODS

The method used at our centre is similar to that described by Mulekar[1] which was a modification of the technique described by Olsson and Juhlin.[2] This report is a retrospective analysis of 58 patients who were operated between December 2003 and August 2006 and were under follow-up for at least 2 years. The duration of the disease varied between 2 and 15 years. At the time of transplantation, all patients were having stable disease for at least 1 year.

### Patient selection

Patients with patches of vitiligo stable for at least 1 year were recruited for transplantation. The criteria of stability were taken as (a) no new vitiligo patches, (b) no extension of existing vitiligo patches and (c) no loss of pigmentation of previously repigmened patches for at least 1 year. When available, previous photographs were compared to look for any increase in the number or size of the patches.

Unstable vitiligo patients, e.g., patients who had noticed increase in their vitiligo patches in the last 1 year, and patients with unrealistic expectations (patients demanding assurance/guarantee that post-procedure, the vitiligo would never recur on the operated patches and/or fresh areas) were excluded.

### Donor site

The lateral aspect of the gluteal region was selected as the donor area. Care was taken to ensure that the donor area had no vitiligo patches. The size of the split-thickness donor skin was taken as one-tenth of the recipient area while dealing with large confluent patches. In cases having multiple scattered small patches, larger donor skin was taken - approximately one-fifth of the recipient area. Under aseptic precautions, a very superficial sample was harvested using a shaving blade held in straight Kocher’s forceps. The donor area was dressed with a liquid paraffin dressing tulle (Fairlee™) and sterile gauze pad.

### Cell separation technique

The cell separation was done under aseptic precautions in a laminar flow bench kept in the operation theatre. The skin sample harvested was transferred to a Petri dish containing 5 ml of the 0.2% w/v trypsin solution, epidermal side facing upwards, and incubated for 45 min at 37°C. After 45 min, the action of trypsin was neutralized with the trypsin inhibitor (Life Technologies, USA).

The epidermis was separated from the dermis and transferred (epidermis) to a test tube containing 2 ml of Dulbecco’s modified Eagle medium: Nutrient Mixture F-12 (DMEM / F-12) medium (Life Technologies) and vortex mixed for 15 s.

**Figure 1:**
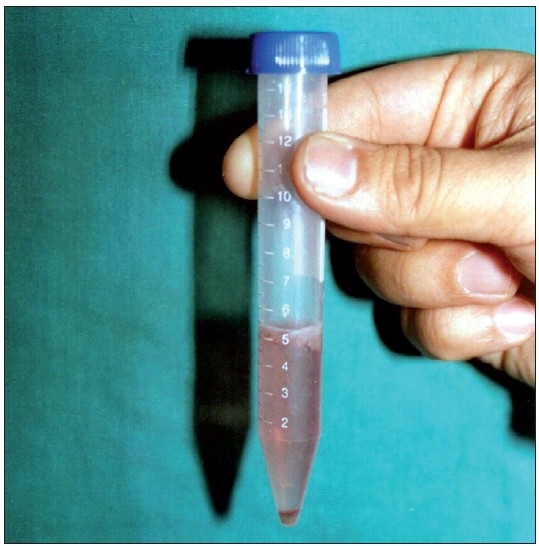
Cell pellet

The epidermis was further broken into smaller pieces in a Petri dish and washed with the DMEM / F-12 medium and finally transferred to a test tube containing the DMEM / F-12 and centrifuged for 6 min. The supernatant was discarded and the pellet was suspended in a test tube [Figure 1]. The final volume prepared varied from 0.2 to 0.5 ml depending on the size of the area to be treated.

### Transplantation technique

**Figure 2:**
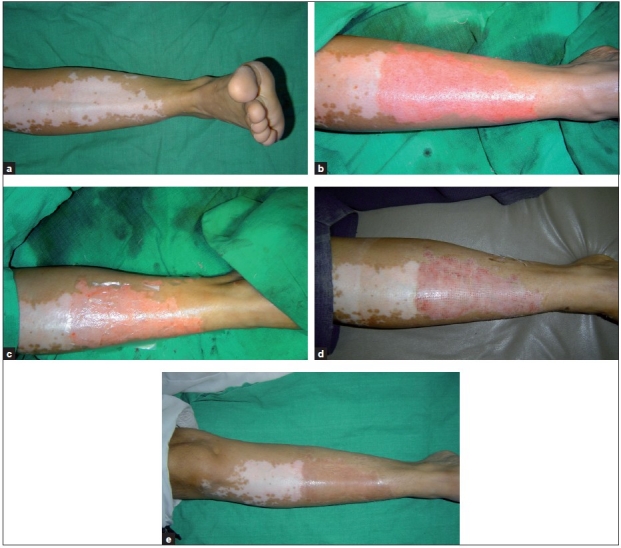
(a) Vitiligo patch on shin; (b) uniform dermabrasion; (c) patch covered with collagen dressing; (d) vitiligo patch on the eighth day after the removal of dressing; (e) uniform pigmentation over the treated area at 3 months

The recipient site was abraded with a dermabrader fitted with a diamond fraise wheel (Delasco™) [Figure 2a and b]. While operating close to the eyelid margins, an Erbium:YAG laser was used with a fluence of 1000 mJ, 1-2 passes. The endpoint of ablation was pinpoint bleeding. Haemostasis was achieved and the ablated area was covered with saline-soaked gauze pieces.

The cell suspension was spread evenly on the dermabraded area and covered with collagen dressing (Collomedica Laboratories) to hold the cells applied [Figure 2c]. This was covered with liquid paraffin and gauze pieces. Patients were instructed to lie still in the same position for at least 1 h to ensure cell fixation and then shifted to a room and further instructed to avoid excessive movements of the treated area for at least 6 h. After this, patients operated under local anaesthesia were permitted to return home. Patients operated under general anaesthesia were admitted overnight and discharged the next day morning.

### Post-procedure instructions

All patients were instructed to take complete rest and avoid all vigorous physical activities. Patients were prescribed oral antibacterial agents for 5 days and non-steroidal anti-inflammatory drugs (NSAIDs) for 3 days. The dressings were removed after 1 week in most cases.

Patients were asked to follow up at weeks 1 and 3, and then at 3-month intervals. Patients were asked to report immediately if they noticed any fresh patches of vitiligo. Patients who had incomplete repigmentation were reoperated after an interval of 6 months only if vitiligo was still stable.

Patients were instructed not to scrub the area and post-procedure no medication was prescribed. Patients were permitted to use make-up on the treated area 10 days after the removal of dressings.

The response to the procedure was graded as excellent if the repigmentation was more than 90%, good if the repigmentation was 70–89%, fair if the repigmentation was 30–69% and poor if the repigmentation was less than 30%.

PUVA or PUVAsol was initiated if there was a delayed onset of pigmentation, if the lesion was appearing hypopigmented or if there were some skipped areas (where pigmentation had not appeared).

## RESULTS

Of the 58 patients operated, 9 patients did not turn up for follow-up after the initially operated areas, e.g., the donor and the recipient area, had healed. The remaining 49 patients were observed for 2 years. The recipient area of most cases epithelialized completely in 7 days [Figure 2d] and no further dressings were usually required. Few areas especially near the ankle required a second dressing which was removed after 3 days by the patient.

The onset of pigmentation was seen earliest at 3 weeks post-operatively; however, in few patients the onset was delayed up to 6 weeks and was evident only after the initiation of psoralen photochemotherapy (PUVAsol or PUVA) or narrow-band ultraviolet B (NB-UVB) therapy. The maximum area operated in one individual patient was 230 cm^2^ and the minimum was 2 cm^2^. Seven patients required a touch-up procedure to cover up the patches which had incomplete repigmentation following the first procedure. This was done at least 6 months after the patient had stopped showing further improvement in spite of receiving phototherapy. In initial few months following the procedure, the treated areas were hypo- or hyperpigmented in many cases, but after 6–8 months they acquired the same colour as the surrounding skin [Figure 2e].

**Figure 3:**
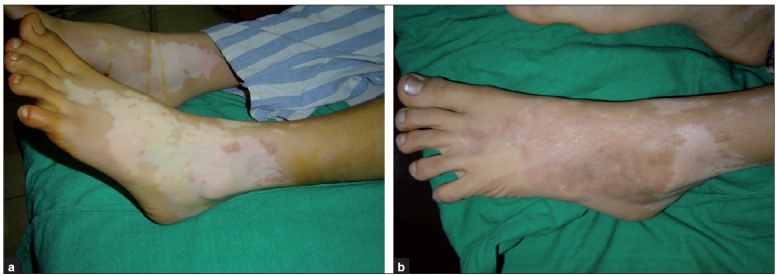
(a) Vitiligo on foot, pre-treatment; (b) uniform pigmentation over the treated area at 3 months

**Figure 4:**
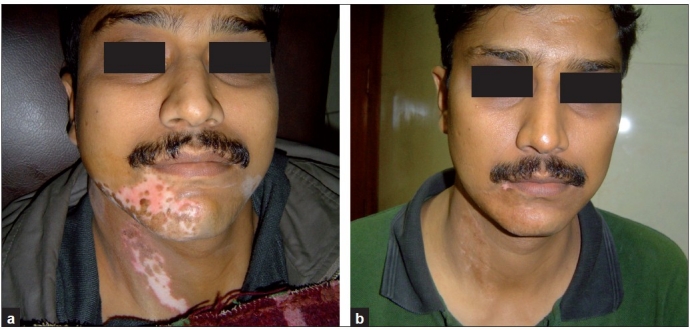
(a) Segmental vitiligo on face and neck, pre-treatment; (b) post-treatment

**Figure 5:**
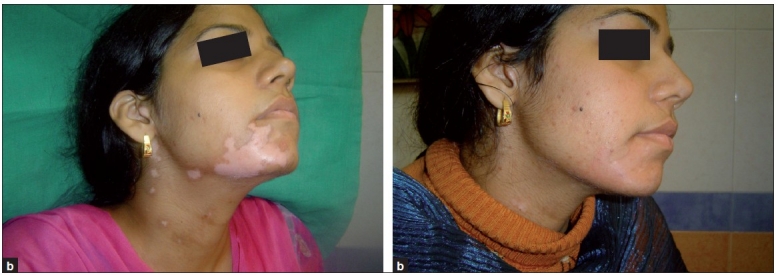
(a) Segmental vitiligo on face, pre-treatment; (b) post-treatment

**Figure 6:**
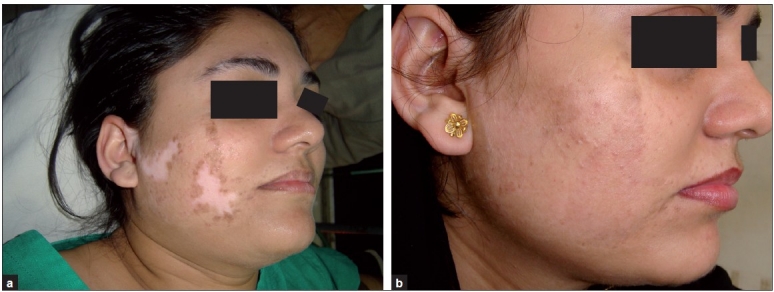
(a) Segmental vitiligo on face, pretreatment; (b) post-treatment

Thirty-two (65%) patients had excellent (>90%) pigmentation [Figures 3–6], 9 (18%) had good (70–89%) repigmentation and 4 patients (8%) each had fair (30–69%) and poor (<30%) responses. Most cases took around 3–6 months for complete pigmentation.

During the follow-up, eight patients had relapse of the disease after 6 months. Of these, five developed fresh lesions while the operated sites were still retaining pigmentation; three patients had loss of pigment over the operated site as well.

Thirty-nine (79%) patients had excellent colour and texture matching; 6 (12%) developed hyperpigmentation and 4 (8%) showed lesional hypopigmentation as compared to the surrounding normal skin. A hypopigmented border was observed in 12 patients.

The donor site repigmented within 1–6 months. In five patients, the donor area healed with hyperpigmentation.

## DISCUSSION

The exact aetiology of vitiligo still remains unclear with various hypothesis, e.g., autoimmune, neural and autocytotoxic mechanisms being proposed. The treatment can be classified into medical treatment, light-based treatment, surgical treatment, camouflage and depigmentation therapy.[3,4]

Medical treatmentincludes the use of immune-modulating drugs such as systemic corticosteroids, levamisole, cyclophosphamide, azathioprine, vitamin supplements (especially vitamin B12 and folic acid).

Light-based treatment includes psoralen photochemotherapy (PUVAsol and PUVA) and NB-UVB, which are usually delivered to the full body, and targeted phototherapy systems which also include excimer laser and excimer lamp.

Surgical treatments can be classified as procedures involving complete skin transfers (e.g., partial split-thickness grafting, punch grafting and blister grafting) and cell transplantations which are further divided into culture and non-culture techniques.[5–7]

Camouflage products include creams and lotions which serve as a temporary make-up. Depigmentation therapy involvestheremoval of pigmented skin in a case of universal, extensive vitiligo.

Vitiligo areas devoid of hair, e.g., finger tips, ankles, dorsum of hand, dorsum of foot, lips, etc., and vitiliginous areas with leukotrichia are resistant to most medical and light-based treatments and hence the replenishment of melanocytes needs to be done surgically to achieve good results.

The goal of all surgical treatments is to obtain complete repigmentation of the vitiliginous areas. An ideal surgical treatment should provide good colour and texture matching of the recipient site with that of the surrounding normal skin. It is also desired that there is no permanent scarring induced at the donor site. Punch grafting is associated with a cobblestone appearance of the grafts and donor site especially seen with bigger punches. Split-thickness grafting may lead to milium formation, thickening of the graft margins, hyperpigmentation or stuck-on appearance in some cases. Also larger sized graft donor sites are required that are at risk for scarring or altered pigmentation.

Recent advances in the surgical methods of treating vitiligo involve the transplantation of cultured pigment cells.[8] This technique involves harvesting of pigment cells from a shave biopsy of the normally pigmented skin in the first step, expanding the cells in culture for about 3–4 weeks, and in the second step transplanting them to an area devoid of pigment cells.[9] The procedure of cell culture has certain limitations such as requirement of elaborate laboratory set-up, risk of contamination during culture and very high costs.

The melanocyte transplantation technique has now been modified to a one-time day care procedure in the form of transplantation of non-cultured melanocyte-keratinocyte suspension. Its advantage is that cell culture is not needed and that skin harvesting from the donor area, preparation of cell separation and application of melanocytes can all be undertaken in a single 3-h procedure.

Hyperpigmentation was observed in cases where proportionally a larger donor area was taken (donor:recipient > 1:5) and in two of these patients the donor area also healed with hyperpigmentation suggesting that the patient had a tendency towards developing post-inflammatory hyperpigmentation. This post-inflammatory hyperpigmentation over the donor and recipient area faded spontaneously over 4-6 months.

Hypopigmentation was observed in areas where large confluent recipient patches were operated and the donor:recipient ratio was more than 1:10; hence the ratio of 1:10 seems ideal for non-cultured melanocyte-keratinocyte transplantation. However, we need to quantify this ratio better so that ideal colour matching can be obtained.

The hypertrophic scar, which was also hyperpigmented, was seen in two cases, one over the ankle and the other over the dorsum of finger. Both the cases had delayed healing probably because these sites were prone to excessive movements.

Eight (16%) patients who were previously stable and had achieved pigmentation relapsed in time varying from 6 months to 2 years. Of these, only three lost pigment from the transplanted site; remaining five patients developed fresh lesions; however, the transplanted areas were spared.

The hypopigmented border around the pigmented patch was observed in 8 (16%) cases, this was more in our initial patients when we were dermabrading only the depigmented area of the vitiligo patch; however, later on while carrying out the dermabrasion 2-3 mm into normal skin, this complication was far less.

The repigmentation of white hair (leukotrichia) was observed in only 3 cases out of 14 and was unpredictable.

## CONCLUSION

Melanocyte cell transplantation is very effective in the treatment of stable, non-progressive vitiligo, the main advantage being that large areas can be treated; with a small donor site with just 8–10 cm^2^ of the donor area, a 100 cm^2^ area of the vitiligo patch can be treated. Repigmentation occurs in most cases within 2–4 months; repigmentation is uniform and matches well with the surrounding skin [Figures 6a–b]. All sites including eyelids, fingers, lips and joints (excluding palms and soles) can be treated.
